# Eustachian Tube Dysfunction Diagnostic Pathway—What Is the Current State of the Art and How Relevant Is Chronic Nasal Disease?

**DOI:** 10.3390/jcm13133700

**Published:** 2024-06-25

**Authors:** Sofia Anastasiadou, Polyzois Bountzis, Dimitrios Evangelos Gkogkos, Petros Karkos, Jannis Constantinidis, Stefanos Triaridis, George Psillas

**Affiliations:** 1 Department of Medicine, Achepa University Hospital of Thessaloniki, Aristotle University of Thessaloniki, 546 36 Thessaloniki, Greece; pkarkos@aol.com (P.K.); janconst@otenet.gr (J.C.); triaridis@hotmail.com (S.T.); psill@otenet.gr (G.P.); 2 Department of Mathematics and Physics, Universita della Campania “Luigi Vanvitelli”, 81100 Caserta, Italy; polyzois1@gmail.com; 3 Department of Applied Informatics, University of Macedonia, 546 36 Thessaloniki, Greece; dgkogkos@uom.edu.gr

**Keywords:** eustachian tube dysfunction, tubomanometry, sonotubometry, chronic nasal disease

## Abstract

**Background**: Eustachian tube dysfunction (ETD) presents a complex diagnostic challenge in otolaryngology, compounded by its multifaceted nature and overlapping symptoms with chronic nasal disease. This article examines the intricacies of ETD diagnosis, emphasising the necessity for a consensus on diagnostic procedures. **Methods**: A review of the literature was performed through the OVID research tool in the Pubmed/Medline databases to identify relevant articles that discuss eustachian tube dysfunction diagnostics as well as its correlation with chronic nasal disease. **Results:** The literature review harvested 201 articles, and only 51 of them were included in the full text review. A consensus statement was identified on eustachian tube dysfunction, function and diagnostics. It appears that there is significant variability in the diagnostic tools used to identify eustachian tube dysfunction. The main diagnostic approaches used are tympanometry, tubomanometry and sonotubometry, combined with the Patient-Reported Outcome Measure ETDQ-7 questionnaire to support the diagnosis of the condition. Nasal pathology is mostly absent from the retrieved studies, while ear pathology is more commonly mentioned in the current literature. **Conclusions**: There is no gold standard diagnostic tool to determine the presence of eustachian tube dysfunction. Further discussion, large multicentre studies and focused research are required to achieve a consensus on a diagnostic approach. The authors suggest a diagnostic pathway that combines subjective and objective diagnostic tools to determine the presence of eustachian tube dysfunction. This pathway is simple and can be used in district ENT departments, highlighting the nasal pathology relevance to ETD.

## 1. Introduction

Eustachian tube dysfunction (ETD) stands as a complex and challenging entity within the realm of otolaryngology, posing difficulties in both understanding its underlying mechanisms and establishing precise diagnostic criteria. As the prevalence of the disease is high and steadily rising [1], it is crucial to establish valid diagnostic criteria. This article aims to shed light on the diagnostic methods used for ETD, especially when there is co-existent chronic nasal disease. The intricate interplay of physiological, anatomical, and functional factors contributes to the complexity of ETD, making its diagnosis and management a difficult goal to achieve. Given the increasing popularity of balloon ET dilation within the field of Ear, Nose, and Throat (ENT) medicine, it is even more crucial to establish a consensus on the diagnostic procedures for the ET [2,3].

This article endeavours to examine the current prevalent approaches mentioned in the literature and underscores the necessity for a well-defined and universally accepted diagnostic pathway. By doing so, patients undergoing assessment before any intervention will benefit from an endorsed diagnostic approach, ensuring that they receive an appropriate treatment plan grounded in evidence. This literature review is conducted to identify the main diagnostic approaches to ETD at present, demonstrate the lack of consensus and highlight the absence of chronic nasal disease correlation with the condition.

## 2. Methods

**Figure 1:**
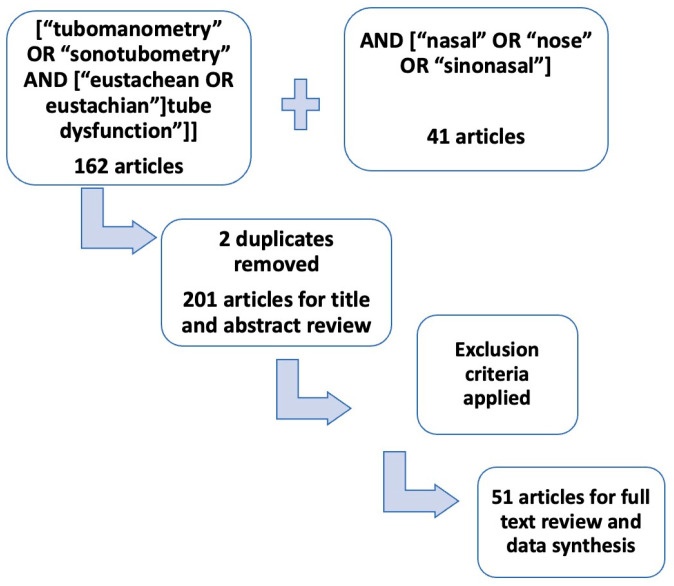
Research process.

A comprehensive review of the literature was performed through the Ovid research tool in the Pubmed/Medline library using the following search terms: [“tubomanometry” OR “sonotubometry” AND [“eustachean OR eustachian”] tube dysfunction”] with an add on search AND [“nasal” OR “nose” OR “sinonasal”]. A separate search in the NICE guidelines was performed to investigate further regarding recent diagnostic guidelines. The first search revealed 162 articles that discuss the diagnostics of ETD, including tympanometry, sonotubometry and tubomanometry. The second search revealed 41 articles, and there was an overlap of 2 articles between the two searches. The titles and abstracts of all 201 articles were assessed. Articles mentioning tubomanometry or sonotubometry only as an investigation before a procedure such as grommets insertion or balloon dilation, not going into detail regarding diagnosis of ETD, were excluded from full text review. Also, articles discussing tubomanometry or sonotubometry diagnostic value for other relevant diseases such as chronic middle ear disease without mentioning ETD were also excluded from the full text review. The literature review focused on case series and prospective and retrospective studies, as well as systematic reviews that included diagnostic approaches for ETD. The research methods and inclusion criteria can be found in Table 1 and Figure 1. A full text review was performed on 51 articles. The data extracted from 51 articles are shown in Table 2. Table 3 shows the relevant literature review results. This article discusses diagnostic methods of ETD mentioned in the literature and considers the strengths and weaknesses of each one of them, always with relevance to nasal disease. 

## 3. Results

The results show a significant variability in diagnostic measures applied for ETD diagnosis. There are no randomised control trials to identify a single diagnostic tool, or large case series on which to base a possible new diagnostic pathway. There is a vast discussion on combinations of objective and subjective tools to determine the presence of ETD with no solid evidence to suggest the superiority of a single diagnostic approach. The evidence regarding diagnosis is equally poor in adult and child populations, with studies of children representing only 20% of all studies included in the literature review. 

The ETDQ-7 questionnaire is the most-used patient-reported outcome measure (PROM) for identifying symptoms of ETD in the current literature. More specifically, the literature review identified 37 articles mentioning ETDQ-7 as the main way of identifying ETD symptoms, which corresponds to 66% of the articles. This is slightly misleading, as ETDQ-7 was originally published and validated in 2012 by McCoul et al., but the literature review includes articles before this date [53]. Looking closer at the articles from 2012 onwards, ETDQ-7 actually represents 85% of the studies. 

**Figure 2:**
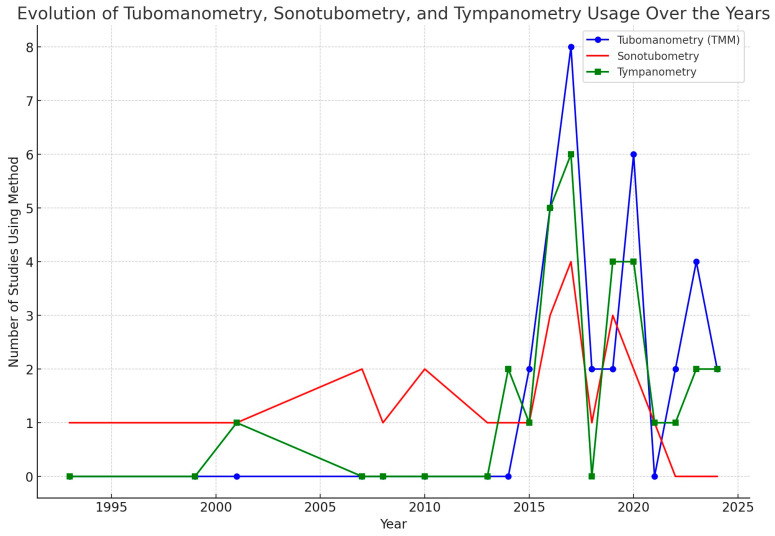
Evolution of ETD diagnostic methods over the years.

In addition, TMM, sonotubometry and tympanometry are mentioned as sole methods or as a combination of investigations in all articles. More specifically, tympanometry is mentioned in 42% of the articles, TMM in 56% and sonotubometry in 47% (Table 2). Please also see the chart in Figure 2. All the articles discuss the diagnostic approaches and mention the lack of a consensus on ETD diagnosis. It is interesting that more recent articles mention TMM much more frequently, while sonotubometry is mostly used in articles before 2010. This might reflect a shift by researchers towards TMM to diagnose ETD, moving away from sonotubometry. 

A study published by Schilder et al. provides consensus on the definition of ET as a pathology, ETD symptoms and diagnostics, while also defining the normal ET function and specifying symptoms and diagnostic tools [54]. The article usefully conveys an agreement on what defines ETD as a medical term. It also describes the presenting symptoms, main diagnostic approaches and possible treatment methods. Another significant guidance was identified in the search of the NICE website regarding balloon dilation of ET and its indications for treatment, which clarifies when balloon dilation is appropriate in ETD management [55]. This guidance is appropriate when assessing patients with ETD before undergoing balloon dilation; however, it does not include diagnostic approaches in cases when an operation is not considered. 

There is limited evidence on the impact of chronic nasal disease on ETD. The current literature review revealed that 25% of the studies correlated ETD with chronic ear disease, while only 8% mentioned chronic nasal disease. Interestingly, all the studies mentioning chronic nasal disease associations with ETD were published the past 5 years, which reflects the increasing interest in this correlation. Up to the present, in the current literature, there is no gold standard diagnostic tool to determine the presence of ETD.

## 4. Discussion

### 4.1. Why Is a Consensus on Diagnostics Difficult to Achieve?

One of the primary hurdles in addressing ETD lies in the multitude of diagnostic considerations [4]. Unlike many medical conditions where diagnostic markers may be clear-cut, ETD often lacks straightforward characterisation [29,56]. The subjective nature of symptoms and the absence of universally agreed-upon diagnostic criteria have led to a diversity of approaches in the clinical setting. As a result, healthcare professionals face a spectrum of diagnostic challenges, ranging from distinguishing ETD from other otologic conditions to defining its severity and impact on patient quality of life [57]. Otological and rhinological conditions that aggravate or even mimic ETD symptoms create more diagnostic uncertainty and lead to the need for a better diagnostic pathway. As mentioned before, another factor aggravating the difficulties encountered in ETD diagnostics is relevant chronic nasal disease, such as nasal congestion due to rhinitis, nasal polyposis or septal deviation that might be causing similar symptoms. Which nasal pathology affects ETD more remains unclear, and if there is any correlation between ETD and chronic nasal disease has not been adequately explored [10,15]. Similarly, chronic ear disease, tympanic membrane retraction and middle ear effusion not relevant to ETD symptoms aggravate the diagnostic nuances and increase the rate of misdiagnosis of the condition.

The consensus by Schilder et al. on ETD definition, types, clinical presentation and diagnosis [54] as well as the National Institute for Health and Care Excellence guidance on eustachian tube balloon dilation [55] are peer-reviewed documents supporting certain diagnostic modalities and treatment approaches in specific cohorts of patients with ETD. Schilder et al. summarised an experts’ panel discussion on ETD, developing a definition regarding what is considered normal function of the ET and concluding that the main diagnostic approaches are otoscopy, otomicroscopy and tympanometry alongside nasal flexible endoscopy. The above are certainly cornerstones for ETD diagnosis; however, the consensus does not mention their limitations, the overlap of findings or further emerging diagnostic tools such as tubomanometry (TMM) and tubosonometry. On the other hand, the NICE guidelines on balloon dilation of ET focus on indications for this treatment modality and not particularly on the broad subject of ETD diagnosis [55]. 

### 4.2. How Do We Diagnose ETD Nowadays?

The diagnosis of ETD often involves the integration of patient-reported outcome tools, yet their reliability can be variable, requiring careful interpretation of the results. Patient-reported outcome measures (PROMs) play a crucial role in capturing the subjective experiences of individuals with ETD, including symptoms like ear fullness, pain, and hearing difficulties [53]. However, the reliability of these tools may be influenced by factors such as individual interpretation, perception, and the subjective nature of symptom reporting. It is essential for healthcare professionals to approach the results of patient-reported outcome tools with caution, considering potential biases and variations in individual experiences. In addition, the PROMs are always developed and applied to a specific population, leaving a significant potential for errors if applied to a different part of the world. It is, therefore, essential to translate, culturally adapt and validate PROMs appropriately before using them as adjuncts for diagnosis [58]. The most broadly used PROM worldwide for ETD is the ETDQ-7 questionnaire, which is a well-established tool for evaluating ETD symptoms, and it has demonstrated its utility in diverse cultural settings [59]. It is considered to reflect ETD complaints and to quantify the most common presenting patient concerns. It has gained prominence for its practicality and reliability in evaluating ETD symptoms [22].

On the absence of symptoms and relevant complaints, there is no indication to proceed to further investigations. If the clinical suspicion emerges from the history taking and/or PROMs results, clinical examination and investigations are warranted. In contemporary clinical practice, a range of diagnostic tests is routinely employed to assess ETD, encompassing tympanometry, audiometry, otoscopy, flexible nasoendoscopy, and various pressure manoeuvres such as Valsalva, Modified Valsalva, Toynbee, Frenzel, or Politzer tests. While these tests provide valuable insights, their lack of unanimous consensus as definitive diagnostic tools underscores the imperative for the exploration of alternative objective measurement techniques. The diversity of available tests highlights the absence of a singular diagnostic tool for conclusively determining the presence of ETD, prompting ENT specialists to explore avenues for innovation [27,29,41,59]. In an effort to augment subjective assessments and enhance the accuracy of ETD diagnosis and management, researchers are actively investigating approaches such as TMM, sonotubometry, or even invasive tubomanometry of the ET. 

### 4.3. Considering the Need for Diagnostic Accuracy, Is TMM the Solution?

In this landscape of diagnostic uncertainty, the need for objective methods becomes paramount. Among the array of diagnostic tools available, tubomanometry (TMM) emerges as a valuable and objective means to assess ET function [9,32]. TMM provides clinicians with a quantitative measure of the pressure changes occurring within the eustachian tube during various manoeuvres, offering insights into its functionality that extend beyond subjective symptom reporting [60]. There are currently only 51 peer reviewed articles that discuss tympanometry, TMM and sonotubometry in the context of ETD diagnosis, and TMM is represented in 56% of them. TMM is considered to aid diagnosis of ETD, which is reflected in its high prevalence in the literature review [32]. The R-value, a parameter derived from TMM, provides meaningful insights into ET function. An R-value ≤ 1 signifies a regular and timely opening of the ET, while an R-value > 1 indicates a delayed opening. The absence of a definable R-value suggests no detectable opening of the ET. This quantitative measure enhances the diagnostic precision in identifying chronic obstructive ET dysfunction [61]. 

However, despite the fact that TMM has been around for a while, it has not gained popularity or wide acceptance, and this is due to a variety of reasons [59,62]. Oehlandt et al. investigated its value in relation to various patient characteristics and concluded it remains a reliable diagnostic tool which is, however, very dependent on patient and operator cooperation [9,63]. In spite of its potential to provide quantitative insights into eustachian tube function, TMM poses significant challenges related to the reliability of results, repeatability, technical intricacies, and patient-specific considerations. The reliability of TMM results is occasionally considered questionable, as variations in operator technique, patient cooperation, and anatomical differences may introduce inconsistencies [14,29]. Reproducibility, a key criterion for any diagnostic method, is not always guaranteed with TMM, leading to potential discrepancies in successive assessments. This lack of repeatability can hinder the method’s utility in tracking changes over time and assessing the effectiveness of interventions.

Furthermore, the technical demands of TMM pose challenges for both practitioners and patients. The procedure requires specialised equipment and a level of expertise, making it less accessible in certain clinical settings. The complexity of performing and interpreting TMM adds an additional layer of difficulty, necessitating skilled personnel for accurate assessments. This complexity may limit its integration into routine clinical practice, particularly in settings where resources and expertise are not readily available. Finally, the cost of obtaining and maintaining the equipment as well as the single-use ear plugs and nose plugs imply high maintenance for this particular piece of equipment. 

Patient factors also play a crucial role in the success of TMM. The method relies on patient compliance and their ability to follow instructions during the procedure. Patients with cognitive impairment, anxiety, or difficulty in understanding and executing instructions may present challenges, potentially compromising the accuracy of the results. Additionally, the need for patients to comprehend and actively participate in the assessment may limit the method’s applicability in certain populations.

However, the efficacy of TMM in clinical diagnostics is notably high, and challenges encountered during the measurement session can be effectively addressed through proper interpretation and, if needed, repeating measurements [32]. To optimise diagnostic accuracy, it is recommended to utilise all three pressures (30, 40, 50 mbar) instead of relying solely on a single, predefined pressure [64]. Recognising that valuable diagnostic information may be lost with a single pressure setting, employing the comprehensive data provided by all three pressures enhances the reliability and thoroughness of the TMM assessment. Talking with the patient beforehand regarding all the steps of the test is very useful for increasing patient understanding and compliance. Mock demonstrations and patient information leaflets are also valuable in this context. With careful interpretation and the flexibility to repeat measurements as required, in experienced hands, TMM emerges as a valuable tool for diagnosing eustachian tube dysfunction and overcoming potential difficulties encountered during the measurement process.

### 4.4. Sonotubometry as a Diagnostic Tool

Sonotubometry, an older diagnostic approach, holds promise for providing valuable insights into ETD and has been tried in the past by Murti et al., Mondain et al. and other researchers with various results [18,19]. The current literature review identified a significant proportion of studies employing sonotubometry to diagnose ETD, with 36% of the studies to incorporating this method in their clinical work up. However, recent studies tend to favour TMM compared to sonotubometry, as shown in Table 3. Researchers have indicated that sonotubometry is very much patient and pathology dependent, and these factors affect correct ETD diagnosis. For example, sonotubometry is challenging in children, and also has heavily altered results when there are middle ear problems. The technique utilises sound waves to assess ET function and, in this way, sonotubometry offers a non-invasive and objective means of evaluating tubal patency and function. It can also be performed with a sealed outer ear canal and a perforated tympanic membrane, increasing the pressure until the ET opens. This measurement reflects the true pressure in the ET. This technique measures the transmission of sound waves through the ET, allowing for the quantification of tube opening and closure dynamics. Indeed, recent studies have demonstrated the potential of sonotubometry to enhance diagnostic accuracy and refine treatment strategies for ETD [38,65,66]. Some articles used prospective studies to reach the above outcome, and others accumulated expert opinions that suggested the above. Larger scale studies supporting sonotubometry are absent from the literature. There are also concerns regarding the efficacy of the method in adults with past ear problems [66,67]. With further research and development, sonotubometry can possibly increase its potential to become a valuable tool in the diagnostic approach to ETD, providing clinicians with a more comprehensive understanding of tubal function and aiding in the optimisation of patient care.

While sonotubometry shows promise as a diagnostic tool for ETD, several challenges have prevented its widespread clinical implementation. One significant difficulty lies in the complex anatomy and dynamic nature of the ET, which can complicate the interpretation of sonometric measurements. Additionally, factors such as variations in patient positioning, anatomical differences, and technical limitations may introduce inconsistencies in sonometric data, potentially affecting the reliability and reproducibility of results. Furthermore, the need for specialised equipment and expertise in conducting and interpreting sonometric assessments poses practical challenges in routine clinical practice. Despite these difficulties, ongoing research endeavours aim to address these limitations and refine sonometric techniques for enhanced diagnostic accuracy and clinical utility [25]. 

### 4.5. What Happens with Diagnosis of ETD with PET or Non-PET?

Distinguishing between patients with and without patulous eustachian tube (PET) holds paramount significance in the realm of otolaryngology and audiology due to the unique clinical manifestations associated with each condition [25,64,68]. In individuals presenting with PET, the ET demonstrates abnormal patency, resulting in symptomatic autophony, audible breathing sounds, and a subjective “popping” sensation [69,70]. Conversely, non-patulous ETD involves obstructive or impaired tubal function, manifesting symptoms such as otalgia, aural fullness and conductive hearing loss with many overlapping symptoms such as “popping” or “crackling” in the ears, tinnitus and the sensation of being underwater.

The precise identification of the specific ET pathology is invaluable in guiding appropriate therapeutic interventions. The current literature review did not focus on diagnostic approaches to discriminate between the two pathologies; nevertheless, concerns are raised regarding the current ETD diagnosis guidance. Management strategies for PET often revolve around mitigating the aberrant patency, whereas non-patulous cases may necessitate interventions focused on resolving obstruction and optimising tube function [71,72]. Establishing a clear diagnostic pathway is imperative for discerning these distinct clinical presentations, enabling targeted interventions that align with the underlying pathology and, consequently, elevating the efficacy of treatment modalities within the specialised field of otolaryngology [73]. There are arguments about the diagnosis of PET with a Cone Beam CT scan, since the bony part of the ET can be adequately assessed on this kind of imaging [74,75]. However, the PET syndrome is multifactorial and the bony part of the ET is not the single pathognomonic finding leading to the diagnosis [74]. There is ongoing research on how to investigate PET syndrome to avoid unnecessary interventions and assess the anatomy appropriately before a symptom relief operation is offered, with dynamic imaging playing an important role towards diagnosis [56,76]. 

In more detail, as previously mentioned, the symptoms associated with PET and non-patulous eustachian tube dysfunction often exhibit significant overlap, rendering them interchangeable and, at times, insufficient for distinctly indicating the underlying pathology [77]. This ambiguity underscores the critical need for diagnostic precision in the evaluation of eustachian tube disorders. The potential interchangeability of symptoms poses a particular challenge, as misidentification can lead to misguided interventions. For instance, interventions designed for the relief of patulous eustachian tube symptoms may exacerbate conditions in non-patulous cases, emphasising the importance of accurate diagnostic methods. Patients with ETD with PET who undergo balloon dilation of their ET are very likely to suffer deterioration of their symptoms as this will enlarge the diameter of their ET, resulting in louder autophony or popping of their ears. A detailed understanding of the presenting symptoms and a meticulous diagnostic approach are imperative to guide appropriate interventions, prevent inadvertent exacerbation of symptoms, and optimise patient outcomes in this complex clinical domain [78].

### 4.6. What Are the Current Views on Diagnostics of ETD in Patients with Chronic Nasal Disease?

Another challenging cohort of patients with ETD present with co-existent chronic nasal pathology associated with ETD symptoms. The diagnostic approach to ETD in patients with chronic nasal disease often overlooks the significance of nasal pathology, despite the evident interplay between the two conditions. In the current literature, Vandersteen et al. shows the significant impact of chronic rhinosinusitis on ETD, while Karaki et al., Kaya et al. and more recently Lima et al. explore and confirm the relationship between ETD and nasal septal deviation [10,79,80]. The current literature review reveals a lack of chronic nasal disease investigations when it comes to ETD diagnosis, while more articles address chronic ear disease. More specifically, only 8% of the studies mention chronic nasal disease while investigating for ETD. In these studies, a comprehensive evaluation commences with a thorough clinical examination encompassing patient history and physical assessment, and the chronic nasal disease aspect is emphasised appropriately. Nasal endoscopy, despite its capacity to reveal nasal abnormalities contributing to ETD, may not consistently receive appropriate attention. Instead, greater importance tends to be assigned to otoscopic findings, particularly the observation of tympanic membrane retraction. Tympanometry, with its ability to identify middle ear pressure abnormalities, often highlights a Type C tympanogram in ETD cases, and it is, therefore, the first diagnostic test when ETD is suspected. If ETD is diagnosed, then chronic nasal disease is considered frequently a separate pathology, which might or might not be treated at the same time as ETD. Therefore, the complicated relationship between chronic nasal disease and ETD may not be fully recognised in the diagnostic process. Recently, there has been an increase in studies that shed light on CRS patients who might demonstrate abnormalities in other parts of their airway epithelium. This might take the correlation of chronic nasal disease and ETD a step further [81]. It is increasingly accepted that either nasal septal deviation or chronic rhinosinusitis may affect the function of the eustachian tube, and the correction of the nasal condition leads to ETD symptom alleviation [10,15,82]. 

### 4.7. A New Suggested Diagnostic Pathway

**Figure 3:**
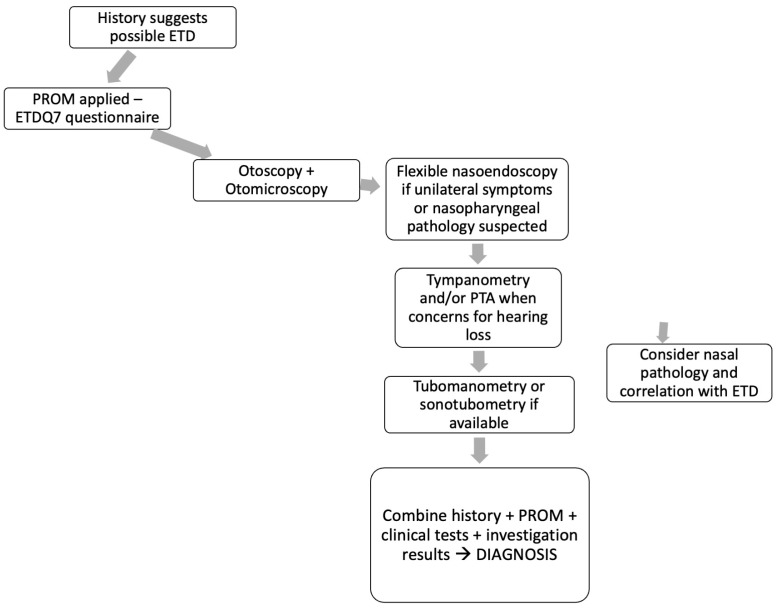
Diagnostic pathway for ETD.

A new diagnostic pathway based on the existing consensus on ET function [54] and NICE guidelines on balloon tuboplasty [55] as well as the current literature review results is suggested to include all the relevant diagnostic tools to achieve ETD diagnosis. The pathway gravitates towards the history and symptoms, ETDQ7 questionnaire and clinical examination findings as well as investigation tools (Figure 3). The complexity of the pathway and the necessity of several steps demonstrates the absence of a single valid test to achieve reliable diagnosis. The new pathway has three main roles. First it combines PROMs, examinations and objective investigations. There are similar pathways described in the literature, but this pathway is redesigned so that it reflects the results of this literature review. Second, it is simple compared to other existing pathways that incorporate expert opinions, CT scans and other investigations. It aims to show that in an ENT department with minimal resources, it is an efficient and valid pathway for assessing ET function. Last but not least, the tab “consider nasal pathology” is innovative as it does not exist in similar pathways. Only very recently, studies are correlating ET pathology with nasal disease, and until now ETD was mostly attributed to middle ear problems. Consequently, this pathway aims to highlight the complexity of the ETD diagnosis and the new emerging factor of chronic nasal disease contributing to this pathology.

## 5. Conclusions

In conclusion, the intricate nature of ETD poses significant challenges to diagnosis and management within otolaryngology. While a variety of diagnostic tests are routinely employed, the absence of a universally accepted gold standard highlights the need for continued exploration of objective measurement techniques. Moreover, the interplay between ETD and chronic nasal diseases adds layers of complexity to the diagnostic process, emphasising the importance of recognising nasal pathology alongside otoscopic and tympanometric findings.

The present literature review demonstrated the need for large studies to ensure a robust diagnostic pathway is formed. At present, all the studies identified use tympanometry, tubomanometry or sonotubometry, combined with PROMs and clinical examination, to establish a diagnosis. Moving forward, it is imperative for healthcare professionals to adopt a comprehensive approach that integrates subjective assessments with objective measurements to achieve a more accurate diagnosis of ETD. 

By acknowledging the various manifestations of ETD and considering the broader context of ear and nasal pathology, clinicians can better understand the underlying mechanisms and tailor treatment strategies accordingly. Ultimately, a holistic approach that accounts for the multifactorial nature of ETD will lead to improved patient outcomes and enhanced quality of care in the management of this challenging condition.

## Figures and Tables

**Table 1:** Inclusion and exclusion criteria.

Inclusion Criteria	Exclusion Criteria
English Language	Studies not mentioning diagnostic approaches
Prospective and retrospective studies, reviews, case series	Studies correlating TMM, sonotubometry or Tympanometry with other diseases
ETD diagnosis/treatment articles	Studies focusing on post-operative outcomes
Consensus papers on ETD	Single case reports

**Table 2:** Basic literature review results—demographics, investigations and other pathology mentioned.

Investigation	Article Representation	%100
Adult studies	41	80%
Children studies	10	20%
ETDQ—7	34	66%
Tympanometry	21	42%
Tubomanometry TMM	28	56%
Sonotubometry	24	47%
Ear pathology mentioned	12	25%
Nasal pathology mentioned	4	8%

**Table 3:** Literature review results.

	Reference	Study (Adult, Children)	PROMs	Objective Method	ETD Diagnosis	Ear Pathology Mentioned	Nose Pathology Mentioned
1	Ikeda et al. (2024) [4]	Adults	Yes	Tympanometry, TMM	Yes	Yes	No
2	Teixeira et al. (2023) [5]	Adults	Yes	TMM	Yes	No	No
3	Bilgili et al. (2023) [6]	Children	Yes	TMM	Yes	Yes	Yes
4	Azevedo C, et al. 2023 [2]	Adults	Yes	Tympanometry, TMM	Yes	No	No
5	Gürtler N, et al. 2024 [3]	Children	Yes	Tympanometry, TMM	Yes	No	No
6	Kaderbay A, et al. 2023 [7]	Adults	Yes	Tympanometry	Yes	No	No
7	Ros L, et al. 2023 [8]	Adults	Yes	TMM	Yes	No	No
8	Oehlandt et al. (2022) [9]	Adults	No	TMM	Yes	No	No
9	Fontes Lima et al. (2022) [10]	Adults	Yes	Tympanometry, TMM	Yes	No	Yes
10	Widodo et al. (2021) [11]	Children	No	Sonotubometry	Yes	No	Yes
11	Jamil et al. (2020) [12]	Adults	Yes	Tympanometry/TMM	Yes	Yes	No
12	Chen et al. (2020) [13]	Children	Yes	Tympanometry/TMM	Yes	Yes	No
13	Ruan et al. (2020) [14]	Adults	Yes	Sonotubometry/TMM	Yes	No	No
14	Vandersteen et al. (2020) [15]	Adults	Yes	Tympanometry/TMM	Yes	No	Yes
15	Manno et al. (2021) [16]	Children	Yes	None	Yes	Yes	No
16	Ma et al. (2020) [17]	Adults	Yes	Tympanometry/TMM	Yes	No	No
17	Ikeda et al. (2020) [18]	Adults	No	Tympanometry/ Sonotubometry	Yes	No	No
18	Joshi et al. (2020) [19]	Adults	No	Tympanometry/TMM, Sonotubometry	Yes	No	No
19	Ikeda et al. (2019) [20]	Adults	No	Tympanometry/ Sonotubometry	Yes	No	No
20	Ikeda et al. (2019) [21]	Adults	Yes	Sonotubometry	Yes	No	No
21	Herrera et al. (2019) [22]	Adults	Yes	TMM	Yes	No	No
22	Zhang et al. (2018) [23]	Adults	No	TMM	Yes	Yes	No
23	Smith et al. (2018) [24]	Adults	Yes	Sonotubometry, TMM	Yes	No	No
24	Schmitt et al. (2017) [25]	Adults	Yes	Tubomanometry	Yes	No	No
25	Takata et al. (2017) [26]	Adults	Yes	Sonotubometry	Yes	No	No
26	Smith et al. (2017) [27]	Adults	Yes	Sonotubometry, TMM	Yes	No	No
27	Leichtle et al. (2017) [28]	Children	Yes	Tympanometry/TMM	Yes	Yes	No
28	Smith et al. (2017) [29]	Adults	Yes	Tympanometry/TMM/Sonotubometry	Yes	No	No
29	Smith et al. (2017) [30]	Adults	Yes	TMM	Yes	No	No
30	Iannella et al. (2017) [31]	Adults	Yes	TMM	Yes	No	No
31	Alper et al. (2017) [32]	Adults	Yes	Tympanometry/TMM	Yes	No	No
32	Xiong et al. (2016) [33]	Adults	Yes	Tympanometry/TMM	Yes	No	No
33	Zhong et al. (2016) [34]	Adults	Yes	TMM	Yes	Yes	No
34	Amoako-Tuffour et al. (2016) [35]	Adults	Yes	Sonotubometry	Yes	No	No
35	Beleskiene et al. (2016) [36]	Adults	No	Tympanometry/Sonotubometry	Yes	No	No
36	Dalchow et al. (2016) [37]	Adults	Yes	Tympanometry/TMM	Yes	No	No
37	Alper et al. (2016) [38]	Adults	Yes	Sonotubometry	Yes	No	No
38	Schroder et al. (2015) [39]	Adults	Yes	TMM	Yes	No	No
39	Jenckel et al. (2015) [40]	Children	Yes	TMM	Yes	No	No
40	Gürtler et al. (2015) [41]	Adults	Yes	Tympanometry/TMM	Yes	No	No
41	Schroder et al. (2014) [42]	Adults	Yes	Tympanometry	Yes	No	No
42	Kitajima et al. (2014) [43]	Adults	No	Sonotubometry	Yes	Yes	No
43	Kitajima et al. (2013) [44]	Adults	No	Sonotubometry	Yes	Yes	No
44	Asenov et al. (2010) [45]	Adults	No	Sonotubometry	Yes	Yes	No
45	Di Martino et al. (2010) [46]	Adults	No	Sonotubometry	Yes	No	No
46	Van der Avoort et al. (2008) [47]	Children	No	Sonotubometry	Yes	Yes	No
47	Takano et al. (2007) [48]	Adults	No	Sonotubometry	Yes	No	No
48	Heerbeek et al. (2007) [49]	Adults	No	Sonotubometry	Yes	No	No
49	Wang et al. (2001) [50]	Adults	No	Tympanometry/Sonotubometry	Yes	No	No
50	Munro et al. (1999) [51]	Children	No	Sonotubometry	Yes	Yes	No
51	Morita et al. (1993) [52]	Children	No	Sonotubometry	Yes	No	No

## Data Availability

All new data discussed in this study are available on the manuscript and further details are available upon request.

## Abbreviations

ET	Eustachian tube
ETD	Eustachian tube dysfunction
PROMS	Patient-reported outcomes
TMM	Tubomanometry
CRS	Chronic rhinosinusitis
NICE	National Institute for Health and Care Excellence
